# Within-Host Competition between Two Entomopathogenic Fungi and a Granulovirus in *Diatraea saccharalis* (Lepidoptera: Crambidae)

**DOI:** 10.3390/insects9020064

**Published:** 2018-06-13

**Authors:** Giuliano Pauli, Gabriel Moura Mascarin, Jørgen Eilenberg, Italo Delalibera Júnior

**Affiliations:** 1 Departamento de Entomologia e Acarologia, Escola Superior de Agricultura “Luiz de Queiroz”, Universidade de São Paulo. Av. Pádua Dias, 11, C.P. 9, CEP 13418-900 Piracicaba, SP, Brazil; giuliano.pauli@fmc.com (G.P.); gabriel.mascarin@embrapa.br (G.M.M.); 2 Embrapa Meio Ambiente, Rodovia SP-340, km 127.5, S/N—Tanquinho Velho, CEP 13820-000 Jaguariúna, SP, Brazil; 3 Department of Plant and Environmental Sciences, University of Copenhagen, Thorvaldsensvej 40, 1871 Frederiksberg C., Denmark; jei@plen.ku.dk

**Keywords:** mixed infection, virulence, *Beauveria bassiana*, *Metarhizium anisopliae*, beta-baculovirus, sugarcane borer

## Abstract

We provide insights into how the interactions of two entomopathogenic fungi and a virus play a role in virulence, disease development, and pathogen reproduction for an economically important insect crop pest, the sugarcane borer *Diatraea saccharalis* (Fabricius) (Lepidoptera: Crambidae). In our model system, we highlight the antagonistic effects of the co-inoculation of *Beauveria bassiana* and granulovirus (DisaGV) on virulence, compared to their single counterparts. By contrast, combinations of *Metarhizium anisopliae* and *B. bassiana*, or *M. anisopliae* and DisaGV, have resulted in additive effects against the insect. Intriguingly, most cadavers that were derived from dual or triple infections, produced signs/symptoms of only one species after the death of the infected host. In the combination of fungi and DisaGV, there was a trend where a higher proportion of viral infection bearing conspicuous symptoms occurred, except when the larvae were inoculated with *M. anisopliae* and DisaGV at the two highest inoculum rates. Co-infections with *B. bassiana* and *M. anisopliae* did not affect pathogen reproduction, since the sporulation from co-inoculated larvae did not differ from their single counterparts.

## 1. Introduction

In nature, infections involving several different categories of invertebrate pathogens may take place in the same invertebrate host and may, through their function, regulate the host population [1,2]. Such interactions allow for studies of a variety of intriguing interactions [3,4,5,6,7].

Hosts represent limited resources; hence, the occurrence of dual or multiple infections leads to within-host competition between invertebrate pathogens [8]. Reports have shown that often, only one insect pathogen is capable of completing its life cycle in the co-infections of a host [9,10,11,12], and the host’s death is commonly attributed to the pathogen that was able to complete its development. Within-host competition between multiple insect pathogens is complex and the outcomes will depend on numerous biological aspects, including the pathogen mode of action, pathogen degree of virulence, pathogen genotype, host age and life stage, host genotype, host carrying capacity, and environmental abiotic conditions. Pathogen interaction outcomes are mostly linked with an increase or a decrease in virulence of one of the involved pathogens [8,13].

Brazil encompasses the largest grown area that is cultivated with sugarcane and it is the first world leader in the exportation of sugar and ethanol [14]. The sugarcane borer, *Diatraea saccharalis* (Fabr., 1794) (Lepidoptera: Crambidae), is the major economically significant pest of sugarcane crops in Brazil, although it has also been recognized as a main pest of corn. Its larval stages are capable of reducing crop yields by feeding on the stalk and thereby facilitating the infection of plant pathogenic fungi through their feeding galleries [15]. Broad-spectrum chemical insecticides have not shown successful results for controlling this insect, because after hatching, the larvae bore the stem and remain hidden until adult emergence, thus creating a difficult target for most insecticides. The two fungal entomopathogens of the most importance to this pest are *Metarhizium anisopliae* (Metsch.) Sorok. (Ascomycota: Clavicipitaceae) and *Beauveria bassiana* (Bals.) Vuill. (Ascomycota: Cordycipitaceae). Both are pathogenic to *D. saccharalis* in its immature stage, and their asexual spores (conidia) germinate and penetrate directly through the host cuticle. Once inside the host, these fungi grow vegetatively in the hemolymph. After a period of time, the host dies by some combination of the depletion of its resources, direct invasion of tissues by hyphae, or the action of pathogen’s toxins, and then the pathogen sporulates shortly after this [16,17]. Both of the fungal species are currently being applied in order to control different insect pests in sugarcane in Brazil, including spittlebugs (*Mahanarva* spp. (Stål) [Hemiptera: Cercopidae]) and weevils (*Sphenophorus levis* Vaurie and *Metamasius hemipterus* L. [Coleptera: Curculionidae]) [18]. The larvae of *D. saccharalis* are also naturally infected by a virus pathogen that, unlike fungi, needs to be ingested. This virus has demonstrated some potential as a biocontrol agent of this pest [19]. This virus belongs to the beta-baculovirus family, genus Betabaculovirus, and is known as Diatraea saccharalis granulovirus (DisaGV). The newly-hatched larvae of *D. saccharalis* become infected when they are fed contaminated leaves with viral particles. The two species of entomopathogenic fungi and the granulovirus exhibit a semelparous life style (*r*-selection strategy), as they release a high number of infective propagules in a single event, which normally coincides with the host death [20]. The larval stages of *D. saccharalis* are exposed to multiple pathogens in the sugarcane agroecosystem, so it is reasonable to suggest that co-occurrence may be recurrent and, thus, makes the within-host competition inevitable. One hypothesis is that the different insect pathogens actually co-exist in *D. saccharalis* populations [21,22]. Based on that, *B. bassiana* and *M. anisopliae* as fungal pathogens and DisaGV as a virus pathogen are appealing as a model system to study for a better understanding of the within-host interactions of pathogens in the sugarcane borer *D. saccharalis*.

The aim of our study was to investigate this complex biological system between one herbivore host and three insect pathogens, by experimentally designing and performing bio-assays, including a host exposure to two or three pathogens at the same time, and measuring the virulence (mortality) and fitness parameters.

## 2. Materials and Methods

### 2.1. Insect Colony

The population of *D. saccharalis* was originally obtained from C.C.A. (Centro de Ciências Agrárias) of the Universidade Federal de São Carlos, and was reared in the Laboratory of Pathology and Microbial Control of Insects at ESALQ/USP. The larvae were fed on 10 mL of an artificial diet [23] inside clear cylindrical glass vials (8.5 × 2.5 cm) (five larvae per vial) at 26 ± 0.5 °C, with 12 h photophase in a growth chamber. The pupae and adults were held in PVC cages and the adults were fed only water. The insect colony was maintained at 25 ± 2 °C with 12 h photophase. For the virulence tests, the larvae from the second egg mass laid were used in bioassays and were always fed an artificial diet.

### 2.2. Entomopathogens

The entomopathogenic fungi that were used in the bioassays included *B. bassiana* (ESALQ-PL63), which was isolated from *Atta* sp. (Hymenoptera: Formicidae) in Piracicaba-SP, and *M. anisopliae* (ESALQ-1037), which was isolated from *Solenopsis* sp. (Hymenoptera: Formicidae) in Porto Alegre-RS. The virus pathogen (Diatraea saccharalis granulovirus [DisaGV]) ESALQ-984, was originally isolated from the *D. saccharalis* larvae in Piracicaba-SP. These entomopathogens were part of the culture collection of the Insect Pathology and Microbial Control Laboratory, ESALQ, University of São Paulo, Brazil, and were stored at −80 °C.

The conidia were transferred to Potato Dextrose Agar (PDA) (Difco^®^, Becton Dickinson, Franklin Lakes, NJ, USA) plates, which were incubated for 10 days in a growth chamber at 26 ± 0.5 °C, and in a 12 h light regime. The conidial suspensions were prepared by scraping the conidia from PDA grown cultures with a sterile loop, and they were then transferred to glass vials containing 15 mL of sterile water, amended with 0.01% surfactant (Tween^®^ 80, Sigma^®^, St. Louis, MU, USA). The conidia concentration (conidia mL^−1^) was enumerated using an improved Neubauer-improved bright line hemacytometer (Hausser-Scientific^®^, Horsham, PA, USA), while the occlusion bodies (OB mL^−1^) of DisaGV were enumerated using a Petroff–Hausser chamber (Hausser Scientific^®^). All of the counts were undertaken at 400× magnification for spores and at 1000× magnification for viral occlusion bodies, under a phase-contrast microscope. The test concentrations were adjusted using the original viral or conidial suspensions to 1 × 10^7^, 5 × 10^7^, 1 × 10^8^, 5 × 10^8^, and 1 × 10^9^ propagules mL^−1^. Additionally, combinations of these entomopathogens, by mixing half of the volume of their respective original suspensions so as to obtain the total final concentrations that have been mentioned above, were also tested. Each entomopathogen was combined with another for dual inoculation, while in the triple inoculation, the three pathogens were mixed together using one third of their volume. The conidial viability was assessed on a PDA plate after 24 h of incubation, at the same conditions above and were ≥95% in all of the cases.

The DisaGV were partially purified from the diseased *D. saccharalis* larvae. The infected larvae were externally sterilized in a solution that contained 2.5% *v/v* sodium hypochlorite and 0.05% (*w*/*v*) streptomycin for 5 min, and were subsequently rinsed with sterile distilled water three times, for 2 min. Afterwards, the larvae were macerated in a buffer solution containing Tris (pH 7.4) and 0.1% (*w*/*v*) sodium lauril sulfate, using a pestle and mortar. Larval debris were filtered through a double layer of cheesecloth and obtaining clear virus suspension full of occlusion bodies. The virus suspension was transferred to a centrifuged Falcon^®^ tube (10 mL per tube) and was centrifuged (Sorvall RT 6000) at 336 g (1500 rpm) and 4 °C for two minutes. The supernatant that contained mostly viral occlusion bodies was retrieved and centrifuged one more time at 5373 *g* (6500 rpm) for 30 min, and at this time, the precipitate was recovered. The viral precipitate was re-suspended in sterile distilled water to form the original, semi-purified virus suspension for use in bioassays. The virus suspension was quantified as it has been indicated above, and averaged 2 × 10^10^ OB mL^−1^. The virus inoculum was maintained at −40 °C until it was used in the experiments.

### 2.3. Virulence Bioassays for Interspecific within-Host Competition

Owing to the large number of treatments, the experiments were repeated five times on different occasions, hence, each treatment had five biological repetitions. The experiments were carried out under controlled environmental conditions in growth chambers that were set to 26 ± 0.5 °C and 12 h light regime. Ten third-instar larvae were tested per concentration, and five biological repetitions per concentration, which totaled 50 larvae per treatment. The treatments consisted of single applications of each pathogen that were tested at all five concentrations (1 × 10^7^, 5 × 10^7^, 1 × 10^8^, 5 × 10^8^, and 1 × 10^9^ conidia or occlusion bodies (OB) mL^−1^) and their respective combinations in pairs or triple. Each experiment included a control group that received only the surfactant solution (0.01% Tween^®^ 80). In total, 8750 larvae were included in the study (i.e., 5 independent experiments × 5 repetitions per treatment × 7 treatments × 5 concentrations × 10 larvae per repetition/treatment).

In the twenty treatments that contained DisaGV, 10 larvae in each arena (1.5 cm height × 6 cm of the diameter plastic plates were fed with 10 small discs of the diet (0.5 × 0.5cm, 0.16 g fresh weight), which had been previously immersed for 10 min in 10 mL of the virus suspensions that were adjusted to the desired concentrations, and were subsequently air dried in a microbiological hood. The diet was prepared without the antimicrobials formaldehyde and methylparahydroxybenzoate (Nipagin^®^, Nutrifarm Distribuidora Ltd.a., São Paulo, SP, Brazil). The larvae were fed contaminated diets with virus occlusion bodies for 36 h prior to being sprayed with fungal suspensions, in order to warrant that the fungal disease did not reduce the dose of virus ingested. Ten larvae separated in five 6 cm plates (arenas) were transferred to a larger plastic Petri dish (140 × 20 mm) and the conidia suspensions were sprayed onto the larvae using a Potter spray tower (Burkard Manufacturing Co. Ltd., Rickmansworth, Hertfordshire, U.K.) at 1 kgf cm^−2^, loaded with 2.5 mL of spore suspension.

The study with only the fungal entomopathogens, which was used to assess their single and combined effects toward *D. saccharalis* larvae, consisted of 16 treatments, and each one was repeated at least five times and 10 third-instar larvae were used per replicate. The treatments consisted of different concentrations of a single fungus or a mixture of both of the fungi, whereas the control group had larvae sprayed only with the surfactant solution (0.01% Tween^®^ 80, Sigma-Aldrich^®^, Germany). After spraying, the larvae remained for one minute on clean and dried plastic plates, prior to incubation.

The larvae were confined in unsealed 60 × 15 mm plastic plates (ten larvae per plate) and were fed daily a fresh diet. The dead larvae were recorded daily over 14 days post-inoculation and were removed from the arenas so as to avoid cross-contamination. The dead larvae that were retrieved from any fungal treatment were transferred to a humid chamber in order to confirm mycosis, while the virus treated larvae were checked for DisaGV infection through observations of the typical symptoms of discolored integument and flaccid body, which was confirmed by the presence of occlusion bodies in the hemolymph samples. In order to avoid sample destruction, the dead larvae from the treatments with both of the inoculations of the virus and fungus were transferred to humid chambers, and only the ones that had not presented fungus mycosis and sporulation were checked for occlusion bodies in the hemolymph. Although a viral infection could have been established by the occlusion bodies that were present inside the cadavers expressing mycosis, none of them resulted in external symptoms of the virus disease.

The dead larvae that were as a result of fungal infection were weighed and then incubated individually in a humid chamber (individually placed in a 60 × 15 mm plastic plate with a moistened cotton wool ball), so as to promote fungal conidiogenesis and to confirm the cause of mortality. After that, the conidial production was determined using a Neubauer chamber and were expressed as conidia per mg of larva, for each of the fungal species. This evaluation allowed us to compare the in vivo pathogen reproduction between a single and dual inoculation of *B. bassiana* and *M. anisopliae*. The entire experiment was repeated twice using different fungal inoculums and larval cohorts.

### 2.4. Statistical Analysis

The concentration–mortality response data, which was accumulated for day 14 post-inoculation, were fitted to a logistic (or logit) regression in a generalized linear model, with a logit link function and binomial distribution for errors. The said model might have been written as follows:

(1) $$y=\frac{1}{1+e^{-}{}^{\left(\alpha +\beta *\log\left(x\right)\right)}}$$

where *x*, *α e β* are the pathogen concentration, intercept, and slope, respectively. The model selection was based on the deviance criterion (given by the ratio of Pearson χ^2^ and residual degrees of freedom of the experiment) and on the likelihood ratio test, compared with other test models, including Gompertz (cloglog) and probit (normal). The logit regression allowed for the estimation of lethal concentrations for all of the pathogen treatments (*LC*_50_ and *LC*_90_) and their respective 95% confidence interval (CI). A heterogeneity factor (h) was calculated by the model, when necessary (i.e., deviance/df > 1), as a means to accommodate the overdispersion parameter [24]. These data were run using the ‘glm’ procedure (generalized linear model) from SAS Institute [25]. The ratio test of the LC_50_s was calculated along with its 95% CI, in order to compare the relative virulence of a pathogen treatment with a standard pathogen, which was usually the one attaining the lowest *LC*_50_ value [26]. Significance for the ratio test was set to *p* < 0.05, and *LC*_50_s were not significantly different if 95% CI of the ratio included 1 [26]. The type of interaction was classified according to the formula that was proposed by [27], which calculated the expected lethal concentration (*LC*_(*m*)_) of a two- or three-component mixture of entomopathogens. For instance, the formula for a three-component mixture was described as follows:

(2) $${LC}_{50(m)}={\left[\frac{r_{a}}{{LC}_{50(a)}}\;+\;\frac{r_{b}}{{LC}_{50(b)}}\;+\;\frac{r_{c}}{{LC}_{50(c)}}\right]}^{-1}$$

where *r_a_*, *r_b_*, and *r_c_* represent the relative proportion of each pathogen that is applied in the mixture, while *LC*_50(*a*)_, *LC*_50(*b*)_, and *LC*_50(*c*)_ are the expected median lethal concentrations of each pathogen alone. The expected *LC*_50(*m*)_ values for multiple pathogen inoculation in the mixture were contrasted with the observed values and their respective 95% CI. If the expected value (*LC*_50(*m*)_) was included within the 95% CI, then it was concluded that the interaction was additive. However, if the expected value was greater than the upper limit of the 95% CI, the interaction was considered synergism. Conversely, if the expected value was smaller than the lower limit of the 95% CI, the interaction was hence scored as antagonism. The interactions were also determined by comparing the concentration–response curves, using a likelihood ratio test, through a process of model simplification, which compared the full model and the reduced models so as to determine the treatment effects (the test of equality was the common slope and intercept) and the concentration by the treatment effects (the test of parallelism was the equal slopes and different intercepts). A significant change in slope, as determined by a significant ‘concentration by treatment’ interaction (hypothesis of different slopes) might have indicated different virulence between the pathogens, whereas a significant treatment effect (hypothesis of equal slopes) was evidence of an agonistic or antagonistic interaction between the two or three pathogens that were applied together [26]. In the same virulence assay, the median survival times (ST_50_) were computed via survival analysis that was performed in SPSS 17.0 software [28], using the non-parametric Kaplan–Meier method for the data that were censored up to 14 days post-inoculation. The estimated ST_50_ values were considered different from each other when their accompanying 95% CI did not overlap.

## 3. Results

**Figure 1:**
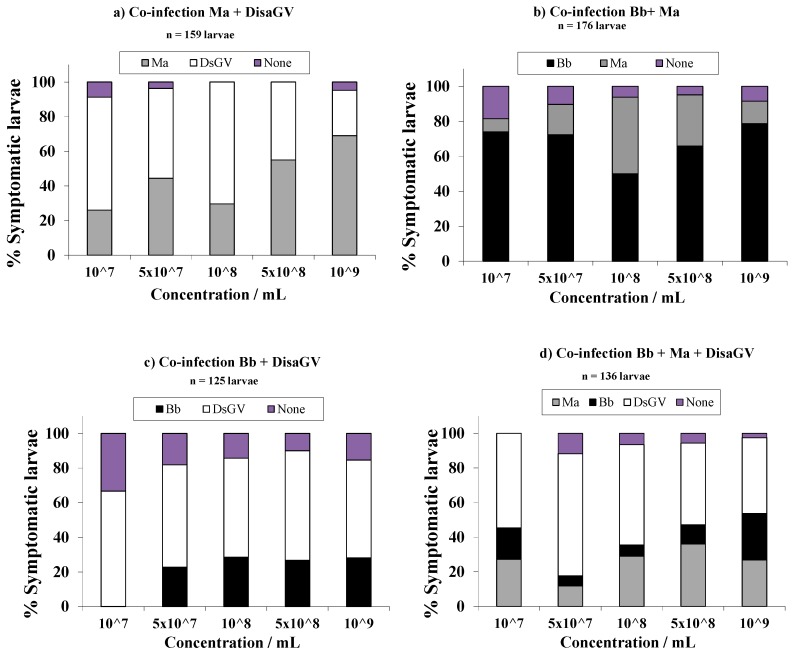
Percent infection attributed to each insect pathogen after a single or combined inoculation of fungal and virus pathogens on the third instar larvae of sugarcane borer after exposure to different concentrations of these pathogens. Number of larvae (n) assayed was reported for each test. Ma—*Metarhizium anisopliae*; Bb—*Beauveria bassiana*; DisaGV—Diatraea saccharalis granulovirus.

Natural mortality in control groups was null in all of the experiments over the 14 day period of incubation, as all of the larvae completed their development and turned into pupae. The inoculum density (concentration–mortality) relationship followed a logistic sigmoidal pattern, with curve steepness increasing with the progression of time (Figure S1 in the Supplementary Material), which indicated that greater mortality levels were achieved with the increase in pathogen density, along with the time after inoculation. The survival analysis evidenced greater larval mortality proportions following the increase in concentration of single or combined applications between the pathogens. With respect to the concentration–mortality relationship, the logistic regression model accurately revealed that the larval mortality was directly and positively related to the concentration of the entomopathogens at all of the single and mixed applications, as supported by the significant slopes (*p* < 0.05) that ranged from 0.95 to 1.54 (Figure 1, Table 1). The *M. anisopliae* showed the steepest slope for the concentration–mortality curve.

Furthermore, the higher inoculum densities of either single or mixed treatments of pathogens were reflected in a faster speed of lethal action as well as decreased ST_50_ values. However, on the basis of these survival curves, the larvae that were inoculated with dual or triple inoculations of pathogens, resulted in a reduction mainly of fungal virulence, as notably represented by the lower ST_50_ values, compared with the larvae that were inoculated with each pathogen separately for most of the concentrations that were tested (Table 2). Compared to the fungal pathogens, the DisaGV alone exhibited higher ST_50_ values.

Single applications of pathogens rendered concentration–mortality curves that were parallel with the common slopes (Table S1, the interactions were not significant, *p* > 0.05), but the curves presented different intercepts (*p* < 0.05), which suggested that *B. bassiana* and DisaGV caused greater initial mortality levels, at lower concentrations than *M. anisopliae*. With regards to the pathogen mixtures, *M. anisopliae* that was applied with either DisaGV or *B. bassiana* rendered improved mortality rates in an additive manner (Table 1, Table S2). By contrast, the resultant combination of DisaGV with *B. bassiana* rendered predominantly an antagonistic effect, as it was also proved by the increased LC_50_ values compared with *B. bassiana* alone (Table 1, Table S2).

In all of the mixed treatments, the percentage of dead insects that did not show signs or symptoms of disease, from any of the pathogens, varied from 3% (*M.a*. + DisaGV) to 15% (*B.b*. + DisaGV). In the mixture that contained *M. anisopliae* and *B. bassiana,* 69% of the larvae were infected with *B. bassiana* compared with 22% that were nfected with *M. anisopliae*, and the former induced more infection rates at inoculum concentrations of 10^7^ and 10^9^ conidia/mL. In the combination of *M. anisopliae* and DisaGV, there was a trend where a higher proportion of viral infection bearing conspicuous symptoms occurred when the larvae were inoculated with the three lowest concentrations, whereas the fungal disease was more prominent at the two highest inoculum rates (Figure 1).

When applying concomitantly *B. bassiana* and DisaGV, 59% of the larvae showed viral disease symptoms compared with 26% that were attributed to fungal disease symptoms, and the viral disease prevailed over the fungal disease in all of the concentrations that were tested. From the larvae that were inoculated with all three of the pathogens (*M.a*. + *B.b*. + DisaGV), 52% showed viral symptoms, 28% were as a result of *M. anisopliae*, and 15% were attributed to *B. bassiana*, with this trend mostly steady throughout the concentrations that were tested, except for the highest concentration, where both fungi had the same mycosis rate.

Those groups of larvae that were inoculated with single treatments of these pathogens presented confirmed mortality with typical symptoms of *M. anisopliae*, *B. bassiana*, and DisaGV, in 99, 100, and 97% of the larvae, respectively (Figure 1).

The sporulated cadavers from the mixed infection that had signs only of *B. bassiana*, produced similar amounts of conidia per mg of larva than its single counterpart (5.8 ± 0.42 × 10^7^ conidia mg^−1^ and 6.24 ± 0.39 × 10^7^ conidia mg^−1^, respectively; *F*_1, 14_ = 0.35, *p* = 0.565). Likewise, the sporulated cadavers from mixed infections containing *M. anisopliae* did not differ in conidia production from its single counterpart (1.07 ± 0.07 × 10^7^ conidia mg^−1^ and 0.93 ± 0.06 × 10^7^ conidia mg^−1^, respectively; *F*_1,18_ = 2.01, *p* = 0.1741). Mycosed cadavers produced on average four times more conidia of *B. bassiana* than *M. anisopliae*, irrespective of single or mixed infection (*F*_3,30_ = 222.28, *p* < 0.0001).

## 4. Discussion

The outcomes from the interaction among the three entomopathogenic microbes that shared the same host insect were highly dependent on pathogens’ initial titer and their mode of action. Sugarcane borer caterpillars that were exposed to mixtures of *M. anisopliae* and *B. bassiana*, *M. anisopliae* and DisaGV, and *M. anisopliae* and *B. bassiana* and DisaGV, exhibited considerably higher mortality rates with the interaction being additive, whilst antagonism was scored when mixing together *B. bassiana* and DisaGV that corresponded to lower virulence factors than their counterparts applied individually. As a result, none of the pathogens’ combinations rendered a synergistic effect. A lack of synergism was more common than it was previously thought, when mixing the distinct pathogens at the same concentration of propagules (half amount of each pathogen), since this combination induced a similar virulence, as they were applied alone. These results were in agreement with [12,29,30], who reported a lack of synergistic interaction for mixing the treatments of pathogens. They also pointed out that the time for death could have taken longer when the most virulent pathogen has its inoculum diluted in mixtures. Varying results are reported in the literature in which additive or synergistic interactions between the pathogens might have been driven by the order of the infection and initial inoculum density that was used [31,32,33]. Other authors observed that synergistic interaction might have happened when applying *M. anisopliae* and *B. bassiana* together on *Schistocerca gregaria* [13]. Our results with *D. saccharalis* revealed that co-inoculation with these fungi did not render increased mortalities. Co-inoculation of larvae with *M. anisopliae* and DisaGV slightly increased the overall virulence, as measured by LC_50_, but not as measured by ST_50_.

Our results revealed that the mixture of *B. bassiana* and DisaGV was antagonistic and opposite to what was found by [22], who observed an increased overall virulence against *D. saccharalis* when applying *B. bassiana*, *B. brongniartii*, and DisaGV together, in comparison with their counterparts alone.

It is noteworthy to point out that only one pathogen from he co-inoculated hosts was able to outgrow and colonize the insect cadaver, rather than more than one pathogen, which indicated that there was always a dominant pathogen from the mixed infections. Similar to our finding here, the majority of studies that dealt with mixed infections in host insects, showed that mostly only one pathogen would dominate the outgrowth after the host’s death [8,10,11,12,29,30,34]. Some authors observed that after mixed infection by *B. bassiana* and *M. flavoviridae* in *Melanoplus sanguinipes*, the former developed quicker in the insect hemolymph than in the latter [11]. Interestingly, among all of the mixed treatments that were tested, we noted that the antagonistic combination between *B. bassiana* + DisaGV induced a greater proportion of host cadavers that were free of colonization (15%), in comparison with the single applications of these pathogens.

The proportion of dead larvae showing DisaGV symptoms seemed to be higher in the mixed infection with the fungi, except for *M. anisopliae*, at the two higher concentrations. This might have been explained by the early inoculation of the virus, as the larvae were fed a diet with DisaGV 36 h prior to the inoculation with the fungi. It was important to point out that we could not diregardc that the cadavers exhibiting fungal mycosis were also infected with DisaGV, with occlusion bodies being present in low numbers in the host hemolymph.

In summary, our results pinpointed the interactions during infection among three important pathogens on *D. saccharalis*. Considering the additive effect of the fungal co-infection of *D. saccharalis*, this might have been an advantage in biological control. The outcome of co-infection by one fungus and DisaGV would depend on the fungal species, and thereby it might have been an advantage or a disadvantage to apply the virus with a fungus.

## 5. Conclusions

Co-inoculation of *Beauveria bassiana* and granulovirus (DisaGV) in *Diatraea saccharalis* larvae resulted in antagonistic effect on virulence, compared to their single counterparts. By contrast, combinations of *Metarhizium anisopliae* and *B. bassiana*, or *M. anisopliae* and DisaGV, have resulted in additive effects against the insect. Most cadavers that were derived from dual or triple infections, produced signs/symptoms of only one species after the death of the infected host. Co-infections with *B. bassiana* and *M. anisopliae* did not affect pathogen reproduction, since the sporulation from co-inoculated larvae did not differ from their single counterparts.

## Figures and Tables

**Table 1:** Estimates of median lethal concentrations (*LC*_50_) and relative potencies by fitting a logistic regression model to the concentration–mortality response data of fungal and virus pathogens, and their combinations against the third instar larvae of sugarcane.

Inoculum	N	df	^a^ χ^2^	Slope ± SE	Intercept ± SE	^b^ h	LC_50_ Observed (95% CI)	^c^ LC_50_ Expected
Ma	300	28	32.41	1.54 ± 0.24 **	−11.9 ± 1.9	1.2	5.61 × 10^7^ (3.1 × 10^7^; 9.1 × 10^7^)	-
Bb	300	28	39.75	1.02 ± 0.24 **	−7.3 ± 1.9	1.42	1.35 × 10^7^ (1.86 × 10^6^; 3.3 × 10^7^)	-
DisaGV	300	28	23.61	1.12 ± 0.20 **	−8.4 ± 1.6	1.0	3.36 × 10^7^ (1.4 × 10^7^; 6.0 × 10^7^)	-
Ma + Bb	300	28	30.12	1.04 ± 0.21 **	−7.5 ± 1.7	1.08	1.40 × 10^7^ (3.1 × 10^6^; 3.1 × 10^7^)	2.17 × 10^7 Additive^
Ma + DisaGV	300	28	29.10	0.95 ± 0.19 **	−7.0 ± 1.6	1.04	2.7 × 10^7^ (7.7 × 10^6^; 5.6 × 10^7^)	4.21 × 10^7 Additive^
Bb + DisaGV	300	28	26.30	1.32 ± 0.21 **	−10.7 ± 1.7	1.0	1.22 × 10^8^ (7.4 × 10^7^; 2.0 × 10^8^)	1.92 × 10^7 Antagonistic^
Ma + Bb + DisaGV	300	28	34.02	1.41 ± 0.24 **	−11.2 ± 1.9	1.22	8.5 × 10^7^ (4.7 × 10^7^; 1.5 × 10^8^)	2.49 × 10^7 Antagonistic^

**^a^** Pearson χ^2^ for the fitted logistic model (logit). **^b^** Heterogeneity factor for the fitted model in relation to the experimental data. **^c^** LC_50_ expected and computed by the model quoted in Tabashnik (1992). ** = Significant at *p* < 0.01. Ma—*Metarhizium* anisopliae; Bb—*Beauveria bassiana*; DisaGV—Diatraea saccharalis granulovirus.

**Table 2:** Virulence of fungal and virus entomopathogens against the third instar larva of sugarcane borer, based on the median survival times (ST_50_).

Treatment		ST_50_ (95% FL) ^b^	χ^2^	*p*-Value
Pathogen ^a^	Concentration			
Ma	10^7^	---	---	---
	5 × 10^7^	---	---	---
	1 × 10^8^	6.5 (5.4; 7.7)	141.10	<0.0001
	5 × 10^8^	6.2 (5.6; 6.8)	73.31	0.2787
	1 × 10^9^	3.7 (3.2; 4.2)	102.96	0.0031
Bb	10^7^	---	---	---
	5 × 10^7^	9.9 (9.1; 10.8)	123.68	<0.0001
	1 × 10^8^	7.6 (6.8; 8.4)	120.01	<0.0001
	5 × 10^8^	7.0 (6.2; 7.9)	190.28	<0.0001
	1 × 10^9^	5.3 (4.7; 6.0)	152.76	<0.0001
Ma + Bb	10^7^	9.6 (8.9; 10.5)	91.33	0.0258
	5 × 10^7^	9.0 (8.1; 10.2)	134.76	<0.0001
	1 × 10^8^	7.1 (6.3; 8.1)	101.97	0.0038
	5 × 10^8^	6.9 (6.3; 7.6)	80.18	0.1296
	1 × 10^9^	5.7 (5.1; 6.2)	169.61	<0.0001
^d^ DisaGV	10^7^	---	---	---
	5 × 10^7^	---	---	---
	1 × 10^8^	10.0 (9.3; 10.7)	98.47	0.0074
	5 × 10^8^	11.0 (10.4; 11.5)	43.45	0.9886
	1 × 10^9^	8.8 (8.4; 9.3)	64.99	0.5468
Ma + DisaGV	10^7^	---	---	---
	5 × 10^7^	11.3 (10.5; 12.3)	68.48	0.4267
	1 × 10^8^	11.6 (10.9; 12.6)	76.93	0.1904
	5 × 10^8^	9.2 (8.5; 10.0)	63.58	0.5957
	1 × 10^9^	7.4 (6.7; 8.2)	217.60	<0.0001
Bb + DisaGV	10^7^	---	---	---
	5 × 10^7^	---	---	---
	1 × 10^8^	10.9 (10.2; 11.7)	110.97	0.0006
	5 × 10^8^	10.6 (9.9; 11.3)	94.46	0.0152
	1 × 10^9^	9.0 (8.3; 9.9)	86.70	0.0531
Ma + Bb + DisaGV	10^7^	---	---	---
	5 × 10^7^	---	---	---
	1 × 10^8^	10.2 (9.4; 11.3)	85.88	0.0599
	5 × 10^8^	7.4 (6.5; 8.5)	150.36	<0.0001
	1 × 10^9^	7.4 (6.8; 7.9)	115.41	0.0002

Ma—*Metarhizium* anisopliae; Bb—*Beauveria bassiana*; DisaGV—Diatraea saccharalis granulovirus.

## Supplementary Materials

The following are available online at http://www.mdpi.com/2075-4450/9/2/64/s1, Figure S1: Three dimension plots describing the temporal progression of sugarcane borer larval mortality as function of the inoculum concentration, when exposed to single or a mixture of two or all three of the pathogens. Right legend represents a gradient of mortality on a gray scale. D.A.A.—days after application; Table S1—pairwise comparisons for logit-lines of single inoculations of fungal entomopathogens and granulovirus, based on tests whether slopes and intercepts are different or not, using the likelihood ratio test. Table S2—pairwise comparisons for single entomopathogen inoculations in contrast with their mixed (combined) applications, based on test hypothesis of parallelism or equality, using the likelihood ratio test.
