# Supplementary material for: Within-Host Competition between Two Entomopathogenic Fungi and a Granulovirus in Diatraea saccharalis (Lepidoptera: Crambidae)
Source: Insects. 2018 Jun 13;9(2):64. doi: 10.3390/insects9020064 (PMC6023289; doi:10.3390/insects9020064)
Supplement: Supplementary file 1 [file insects-09-00064-s001.pdf]

Table S1. Pairwise comparisons for logit-lines of single inoculations of fungal entomopathogens and granulovirus based on tests whether slopes and intercepts are different or not, using the likelihood ratio test. .

Pairwise comparison	Pathogen effect			Concentration effect			Pathogen × Concentration effect		
	deviance	df	adj. *P**	deviance	df	adj. *P**	deviance	df	adj. *P**
Bb *vs*. Ma	52.92	2, 48	**< 0.001**	79.86	1, 47	**< 0.001**	2.93	1, 46	0.087
Bb *vs*. DisaGV	60.487	2, 48	**< 0.001**	57.36	1, 47	**< 0.001**	0.128	1, 46	0.72
Ma *vs*. DisaGV	27.70	2, 48	**< 0.001**	88.95	1, 47	**< 0.001**	1.93	1, 46	0.16

*P-values adjusted for three pairwise comparisons with Bonferroni. Significant *P*-value is highlighted in bold.

Ma = *Metarhizium anisopliae*

Bb = *Beauveria bassiana*

DisaGV = Diatraea saccharalis granulovirus

Table S2. Pairwise comparisons for single entomopathogen inoculations in contrast with their mixed (combined) applications based on test hypothesis of parallelism or equality, using the likelihood ratio test.

	Pathogen × Concentration effect (parallelism test)			Pathogen effect (equality test)		
Pairwise comparison	deviance	df	adj. *P**	deviance	df	adj. *P**
Bb *vs*. "Bb + Ma"	0.005	1, 47	1.00	0.003	2, 48	1.00
Bb *vs*. "Bb + DisaGV "	0.751	1, 47	0.833	9.20	2, 48	**< .0001**
Bb *vs*. "Bb + Ma + DisaGV "	1.083	1, 47	0.393	5.54	2, 48	**< .0001**
Ma *vs*. "Ma + Bb"	1.927	1, 47	**0.014**	3.57	2, 48	**< .0001**
Ma *vs*. "Ma + DisaGV "	3.042	1, 47	**< .0001**	1.823	2, 48	**0.021**
Ma *vs*. "Ma + Bb + DisaGV "	0.122	1, 47	1.00	0.76	2, 48	0.824
DisaGV *vs*. "DisaGV + Bb"	0.445	1, 47	0.997	5.05	2, 48	**< .0001**
DisaGV *vs*. "DisaGV + Ma"	0.343	1, 47	1.00	0.175	2, 48	1.00
DisaGV *vs*. "DisaGV + Bb + Ma"	0.77	1, 47	0.812	2.27	2, 48	**0.003**

[*]*P*-values adjusted for nine pairwise comparisons with Bonferroni. Pathogen applied alone (single inoculation), to which all other mixed treatments (inoculations) are compared according to the likelihood ratio test at $P < 0.05$ (significant *P*-value is highlighted in bold).

Ma = *Metarhizium anisopliae*

Bb = *Beauveria bassiana*

DisaGV = Diatraea saccharalis granulovirus

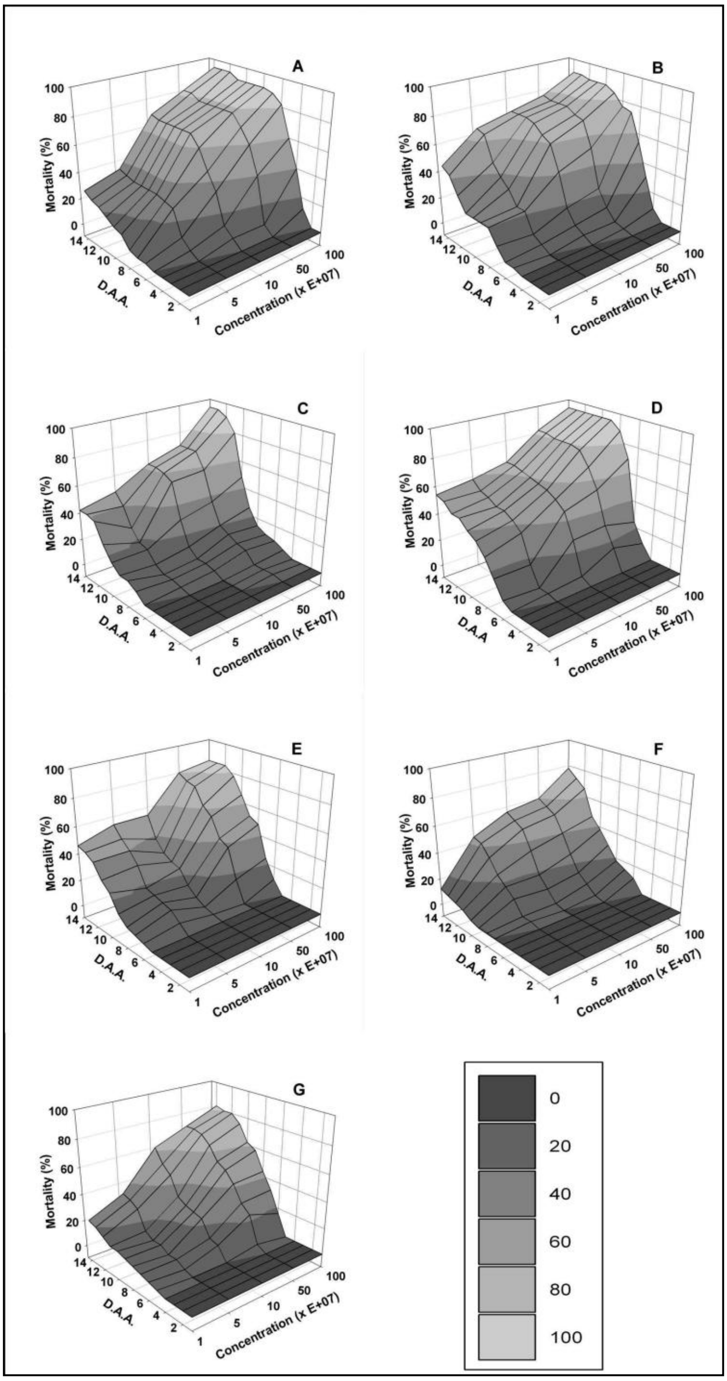

Figure S1. Three dimension plots describing the temporal progression of sugarcane borer larval mortality as function of the inoculum concentration when exposed to single or a mixture of two or all three pathogens. Right legend represents a gradient of mortality on a gray scale. D.A.A. = days after application. A) *M. anisopliae* alone, B) *B. bassiana*

alone, C) DisaGV alone, D) *M. anisopliae* + *B. bassiana*, E) *M. anisopliae* + DisaGV, F) *B. bassiana* + DisaGV, G) *M. anisopliae* + *B. bassiana* + DisaGV.
